# Intrinsic Dissolution Rate Profiling of Poorly Water-Soluble Compounds in Biorelevant Dissolution Media

**DOI:** 10.3390/pharmaceutics12060493

**Published:** 2020-05-28

**Authors:** Alexandra Teleki, Olivia Nylander, Christel A.S. Bergström

**Affiliations:** 1 Science for Life Laboratory, Department of Pharmacy, Uppsala University, Uppsala Biomedical Center P.O. Box 580, SE-75123 Uppsala, Sweden; 2 Department of Pharmacy, Uppsala University, Uppsala Biomedical Center P.O. Box 580, SE-75123 Uppsala, Sweden; olivianylander@gmail.com; 3 The Swedish Drug Delivery Center, Department of Pharmacy, Uppsala University, Uppsala Biomedical Center P.O. Box 580, SE-75123 Uppsala, Sweden

**Keywords:** intrinsic dissolution rate, biorelevant dissolution media, FaSSIF, FeSSIF, poorly water-soluble drugs, physicochemical properties, controlled suspension

## Abstract

The intrinsic dissolution rate (IDR) of active pharmaceutical ingredients (API) is a key property that aids in early drug development, especially selecting formulation strategies to improve dissolution and thereby drug absorption in the intestine. Here, we developed a robust method for rapid, medium throughput screening of IDR and established the largest IDR dataset in open literature to date that can be used for pharmaceutical computational modeling. Eighteen compounds with diverse physicochemical properties were studied in both fasted and fed state simulated intestinal fluids. Dissolution profiles were measured in small-scale experimental assays using compound suspensions or discs. IDR measurements were not solely linked to API solubility in either dissolution media. Multivariate data analysis revealed that IDR strongly depends on compound partitioning into bile salt and phospholipid micelles in the simulated intestinal fluids, a process that in turn is governed by API lipophilicity, hydrophobicity, and ionization.

## 1. Introduction

Pharmaceutical profiling of candidate drugs has primarily focused on properties, such as permeability and thermodynamic equilibrium solubility, as these are used to categorize them according to the biopharmaceutics classification system (BCS) [1]. Many active pharmaceutical ingredients (API) suffer from dissolution rate- and solubility-limited absorption and are categorized as BCS class II (low solubility but high intestinal permeability) or IV (low solubility and low permeability) compounds. In fact, the drug discovery and development pipeline of the pharmaceutical industry is estimated to comprise at least 50–60% of BCS class II compounds [2]. Formulation strategies aimed at improving dissolution of these compounds are often guided by measurements of their intrinsic dissolution rates (IDR, μg/min/cm^2^), i.e., the surface specific dissolution rate of the compound [3]. IDR is an intrinsic property of the compound that can indicate selection of a certain solid state form (e.g., salt, co-crystal, or polymorph) [4], enabling formulation strategy (e.g., solid dispersion or cyclodextrin inclusion) [5,6], or optimize particle size to ensure complete dissolution of a certain drug dose during intestinal transit [7].

Typically, only a few milligrams of API are available to formulation scientists during early drug development. Therefore, much research effort over the past two decades has been devoted to developing miniaturized experimental assays that allow for high throughput measurements of solubility and dissolution rate using small amounts of starting material. The main approaches and equipment available are (i) miniaturized dissolution vessels, flow-through cells, or microtiter plate-based methods with off-line determination of API concentration changes [8], (ii) imaging-based methods [9], and (iii) small-scale (sometimes) automated dissolution instruments with *in situ* API concentration determination by UV fiber optic probes [10,11,12,13]. The interested reader is referred to a recent review for a comprehensive description of all methods relevant for IDR determinations [3]. Here, we will focus on the third method, the UV fiber optic probes. When used *in situ* such probes can monitor drug concentration over time during dissolution by UV absorbance spectroscopy. This method is particularly well suited to study the early stages of dissolution, as absorbance spectra can be collected very frequently (every second). Furthermore, high sample throughput can be achieved by running several assays in parallel without the need for off-line analytics. Commonly used, commercially available equipment includes the μDiss Profiler, SiriusT3, and inForm instruments (Pion Inc., Billerica, MA, USA). For example, the μDiss Profiler measures, e.g., solubility for up to eight samples in parallel, while the SiriusT3 and inForm instruments are fully automated with autotitration and sample autoloader modules. Sample amount ranges from 5–10 mg (μDiss Profiler and Sirius T3) up to 100 mg in the inForm with dissolution media volumes in the range of 3–80 mL. In comparison, traditional determination of IDR (e.g., USP 1087) is carried out in six-vessel US Pharmacopeia (USP)-specified dissolution baths containing 900 mL of dissolution media and 150–700 mg of pure API compressed into discs, and relies on sampling and off-line API concentration determination by, e.g., high-performance liquid chromatography (HPLC).

Measurement throughput for IDR screening is further governed by the time required for API sample preparation and choice of dissolution assay method. Traditionally, i.e., as described in USP 1087, API is compacted into discs that can be measured in rotating or stationary assemblies immersed in dissolution medium in the USP vessels (Wood’s method [14]). The disc method is also compatible with small-scale dissolution assays. An important prerequisite and assumption here is that the disc surface area remains constant during the entire dissolution experiment. Dissolution measurements from discs typically take several hours because of the small surface area (e.g., 0.071 cm^2^ in the μDiss Profiler) available for dissolution. This limitation can be overcome by using other methods, such as powder- or suspension-based assays, because these methods expose a significantly higher total surface area of the dissolving material than the disc-based method, resulting in roughly 100-fold shorter measurement times than discs [13]. In the powder-based assay, coarse powder is added directly to the vials used for the dissolution experiment, and the apparent total surface area of the compound can then be back-calculated after dissolution rate has been determined [10,15]. In contrast, in suspension-based assays, the particle surface area is determined prior to the dissolution experiment, for example by dynamic light scattering (DLS), and the suspension method provides a more accurate measure of total surface area than the powder method [7,16]. Furthermore, suspensions can be (and typically are) stabilized by polymers or surfactants to hinder sedimentation, which is a phenomenon often observed in powder-based assays [10]. Several studies have demonstrated good agreement of IDR values determined by disc- (DIDR), powder- (PIDR) or suspension-based (SIDR) assays [7,13,17], so the choice of method is primarily governed by the solubility of the studied API. It has been recommended to use discs for S_app_ (apparent solubility) > 1 mg/mL, whereas powder or suspension are used for S_app_ < 100 μg/mL. All three methods can be used for compounds with intermediate solubility (100 µg/mL–1 mg/mL) [17].

However, despite these important advances in small-scale dissolution profiling assays, the underlying physicochemical properties governing IDR of an API are still poorly understood. To better understand which molecular descriptors predict dissolution rate, a large dataset of IDR values of physicochemically diverse compounds has to be established that could ultimately also be used as an *in silico* predictive tool of IDR. To be physiologically relevant, these IDR values have to be collected in biorelevant media that simulate the composition of the intestinal fluids in the fasted (FaSSIF; SSIF = simulated small intestinal fluid) and fed states (FeSSIF) [18]. Such data sets are suitable to be established with small-scale dissolution studies using *in situ* UV probes, ideally from suspension-based assays to increase throughput and reduce material needed. However, previous such studies have only explored a small data set with a maximum of six compounds in FaSSIF and phosphate buffer [7,17].

The aim of this study was therefore to establish a standardized protocol for determining IDR from discs or suspensions (depending on compound solubility) in such simulated intestinal media. We based our study on Andersson et al. [7], who used controlled suspensions to measure IDR, but we simplified the procedure to allow faster preparation of suspensions and thereby greater throughput. This allowed us to establish an IDR database of 18 compounds in four different, commercially available biorelevant media and to use those data for computational modeling to identify correlations between physicochemical properties and resulting IDRs.

## 2. Materials and Methods

### 2.1. Materials

Active pharmaceutical ingredients (APIs), were selected from previous studies performed in our laboratories [7,15,19,20]. Most of the compounds have poor water solubility and belong to BCS Class II [1]. In total, six acids, five bases, and seven nonionizable, the latter with no net charge in the pH interval studied and denoted neutral in the following, were studied (Table 1). Molecular descriptors (at pH 5 and 6.5) of all compounds were calculated with ADMET Predictor 9.0 (SimulationsPlus, Lancaster, CA, USA). All compounds were purchased from Sigma Aldrich (St. Louis, MO, USA) with the exceptions of carvedilol (H. Lundbeck A/S, Valby, Denmark), felodipine (AstraZeneca, Mölndal, Sweden), fenofibric acid (APIChem, Hangzhou, China) and danazol (Toronto Research Chemicals, Inc., Toronto, Canada). All compounds were used as received. FaSSIF and FeSSIF were prepared using powders from Biorelevant.com Ltd. (London, United Kingdom). Two versions of their biorelevant media were tested, FaSSIF-V1 and FeSSIF-v1 (both from FaSSIF/FeSSIF/FaSSGF powder), as well as FaSSIF-V2 and FeSSIF-V2, respectively (Table 2). Buffer solutions for the biorelevant media were prepared with sodium hydroxide pellets (anhydrous, ≥98%), maleic acid (≥99%) and acetic acid (≥99.8%) from Sigma Aldrich, and sodium dihydrogen phosphate monohydrate and sodium chloride from MERCK (Darmstadt, Germany). Stock solutions of API compounds were prepared in dimethyl sulfoxide (DMSO; ≥99%, Sigma Aldrich). The suspension medium contained hydroxypropyl methylcellulose (HPMC; Shin-Etsu Chemical Co., Ltd., Tokyo, Japan), sodium dodecyl sulfate (SDS; ≥98,5%) and polyvinylpyrrolidone K30 (PVP K30) from Sigma Aldrich.

### 2.2. Solubility Determination

Apparent solubility (S_app_) of all compounds except naproxen and ethinylestradiol in both FaSSIF-V1 and FeSSIF-V1 media were obtained from the literature (Table 3). If literature values were not available, S_app_ values were estimated from the dissolution experiments either from the steady state concentration reached during dissolution making use of an excess amount material, or by nonlinear regression analysis of the dissolution data in GraphPad Prism 7.04 (GraphPad Software Inc., San Diego, CA, USA). However, the solubility of naproxen in FaSSIF-V1 and FeSSIF-V1 and ethinylestradiol in FaSSIF-V1 were determined by a modified shake-flask method [21] (as their dissolution was measured from discs). In these experiments, an excess amount of drug was added to triplicate Eppendorf tubes containing 1 mL of the respective dissolution medium. The tubes were vortexed and placed in a 37 °C incubator. Samples for HPLC analysis were taken 24, 48, and 72 h (triplicates at each time point) after initiation of the experiment to ensure that equilibrium had been reached. The samples were centrifuged at 37 °C, 23,000 g for 10 min (Heraeus Megafuge 8R, Thermo Scientific, Waltham, MA, USA). The supernatant was sampled, diluted 1:1 with acetonitrile, and kept refrigerated until HPLC analysis. The concentrations of the dissolved drugs were determined using a HPLC (Agilent Technologies 1290 Infinity, Santa Clara, CA, USA) with a Zorbax Eclipse XDB-C18 column (4.6 × 100 mm; Agilent Technologies, Santa Clara, CA, USA) kept at 40 °C. The injection volumes were 20 μL. A mobile phase 0.1% formic acid in acetonitrile: 0.1% formic acid in water 60:40 (v/v) and an isocratic flow rate at 1 mL/min were used for both naproxen and ethinylestradiol. UV absorbances were monitored at a wavelength of 232 nm for naproxen and 315 nm for ethinylestradiol. The retention time was 1.76 min for naproxen and 1.85 min for ethinylestradiol.

### 2.3. Suspensions

#### 2.3.1. Preparation

Controlled suspensions of the APIs were prepared by tip ultrasonication in suspension media. For compatibility reasons [7], the suspension medium composition was 2% PVP K30 and 0.2% SDS in MilliQ water for acidic and neutral compounds or 1% HPMC and 0.2% SDS in MilliQ water for basic compounds. Suspension media were stirred for at least an hour until a clear solution was obtained. All suspensions were prepared at a concentration of 4 mg API/mL suspension medium by weighing 16 mg of the compound into a glass vial and adding 4 mL of the corresponding suspension medium. The suspension was then briefly vortexed prior to 5 min of tip ultrasonication using a Vibra-Cell sonicator mounted with a 13 mm (Ø) probe tip (Sonics, Newtown, CT, USA). The following settings were used: 20% amplitude, pulse 10 s on and 5 s off. The vial containing the suspension was placed in a beaker with ice during ultrasonication to avoid heating. Felodipine and griseofulvin were treated slightly differently to avoid forming a large fraction of submicron-sized particles. The felodipine suspension was only ultrasonicated for 1 min, and the griseofulvin suspension was used immediately after vortexing and without ultrasonication. All prepared suspensions were kept in an oven at 37 °C prior to use in the dissolution experiments. Dissolution experiments were performed within three hours of sample preparation.

#### 2.3.2. Characterization

The freshly prepared suspension was diluted (100 µL in 1 mL MilliQ water) and immediately used for determination of hydrodynamic particle size by dynamic light scattering (DLS; Litesizer 500, Anton Paar GmbH, Graz, Austria) at 25 °C. Peak values from the intensity-based particle size distribution were used for calculating total particle surface area in the suspension following the approach of Andersson et al. [7]. In case of bimodal particle size distributions, an intensity peak weighted average particle size was calculated. The following assumptions were made for the surface area calculations: (i) spherical particles, (ii) constant particle size, and (iii) constant number of particles during the initial phase of the dissolution experiment (i.e., under sink conditions or <0.3 × S_app_). Using these assumptions, the volume of each particle (*V_particle_*) is:

(1) $$V_{particle}=\frac{4\pi r^{3}}{3},$$

where *r* is the mean radius of the suspended particles. The particle surface area (*SA_particle_*) is:

(2) $$SA_{particle}=4\pi r^{2},$$

and the volume of compound (*V_material_*) added to the dissolution medium is:

(3) $$V_{material}=\frac{m}{\rho },$$

where *m* is the total mass of compound added and *ρ* is the density of the compound. The total number of particles (*n*) in the suspension can thus be calculated from:

(4) $$n_{particles}=\frac{V_{material}}{V_{particle}}$$

Finally, the total surface area (*A*, cm^2^) of all particles present in the suspension added to the dissolution medium is calculated through [7]:

(5) $$A=n_{particles}SA_{particle}$$

The powder X-ray diffraction (XRD) measurements of the as-received compounds and suspensions were performed with a Rigaku MiniFlex (1.5406 Å Cu Kα1 radiation; step size 0.01°; Tokyo, Japan). Suspensions were centrifuged for 15 min at 21,000 g (Heraeus Megafuge 8R, Thermo Scientific, Waltham, MA, USA) and the supernatant removed prior to XRD analyses. Compound melting point (T_m_) and heat of fusion (ΔH_f_) were measured by modulating differential scanning calorimetry (DSC; Q2000 DSC, TA Instruments Co., New Castle, DE, USA). Samples (1–5 mg) were weighed into aluminum pans and sealed with a non-hermetic lid. The heating rate was 3 °C/min up to a temperature 20–30 °C above the compound T_m_ with a temperature modulation of ±1 °C every 60 s. The reported T_m_ is the temperature at onset of melting.

### 2.4. Small-Scale Dissolution Measurements

Dissolution experiments were carried out in a small-scale dissolution apparatus using *in situ* fiber optic probes to measure the amount of drug dissolved over time by UV absorbance (μDiss Profiler, Pion Inc., Billerica, MA, USA) [3]. Drug dissolution was carried out from suspensions (S) or discs (D) depending on the solubility of the compound in the biorelevant media studied (Table 3). In most cases, we applied the recent recommendation to use the suspension method for compounds with solubility <100 μg/mL [17], whereas the disc method was used for compounds with higher solubility.

#### 2.4.1. Standard Curve

Standard curves were established by adding aliquots of DMSO stock solutions of the compounds to 3 mL of biorelevant medium, respectively. Dissolution media were prepared following the protocol provided by Biorelevant.com Ltd. and used within 48 h after their preparation. The composition of the four different biorelevant media used in this study is shown in Table 2. The media were incubated at 37 °C for at least one hour prior to use in the dissolution experiments. The hydrodynamic diameter of the micelles/vesicles formed in the biorelevant media were measured by DLS (Table 2). Appropriate UV probe tips for the µDISS experiment were selected based on expected solubility and the strength of the chromophore of each compound; typically, the probe tips used were 10 or 20 mm for the poorly water-soluble compounds. The biorelevant dissolution medium was maintained at 37 °C in the μDiss Profiler and stirred at 800 rpm during addition of the DMSO stock aliquots. Stirring was turned off while measuring the UV absorbance for each aliquot and the standard curve was established based on six aliquots.

#### 2.4.2. Dissolution from Controlled Suspensions

Glass vials (six in parallel), each containing 15 mL of biorelevant dissolution medium and a crossbar stirrer, were used for the dissolution experiments. The stirring rate was set to 100 rpm. Freshly prepared API suspensions were briefly vortexed to re-disperse any sediments and 500 μL of suspension was added to each vial to initiate the experiment. The temperature was kept at 37 °C and the same volume of suspension was added for all compounds regardless of their solubility values in the media used, with the purpose to establish a rapid and functional measurement protocol. Experiments were run for at least 20 min with data collected every 1 s for the first 8 min and then once per minute for the remaining time. All dissolution experiments from suspensions were carried out in triplicate.

#### 2.4.3. Dissolution from Discs

Miniaturized discs with a surface area of 0.071 cm^2^ were prepared with a Mini-IDR compression system (2 min at 7 bars; Heath Scientific, Milton Keynes, UK) using approximately 5 mg of compound [11]. The discs were inserted into rotating disc carriers and placed in the vials of the μDiss Profiler. Experiments were initiated by the addition of 15 mL of biorelevant medium. The stirring rate was 100 rpm and the temperature was kept at 37 °C. Data were collected for several hours due to the significantly slower compound dissolution from discs compared to suspensions. All dissolution experiments from discs were carried out in triplicate.

#### 2.4.4. Calculation of the Intrinsic Dissolution Rate (IDR)

The Suspension Intrinsic Dissolution Rate (SIDR, µg/cm^2^min^−1^) was calculated by the following equation:

(6) $$SIDR=V\;k\;\frac{1}{A},$$

where *k* is the initial slope of the *dC*/*dt* curve (concentration in μg/mL per time unit), *V* is the volume of the medium (mL), and *A* is the total particle surface area (cm^2^). The total particle surface area for each suspension used was calculated from the respective DLS measurements (Equation (5)). Total volume was 15.5 mL (15 mL biorelevant dissolution medium with 500 μL suspension added). The initial part of the dissolution curve was used to calculate *k*.

The Disc Intrinsic Dissolution Rate (DIDR, µg/cm^2^min^−1^) was calculated by the following equation:

(7) $$DIDR=\frac{dm}{dt}\;\;\frac{1}{A_{disc}}=V\;k\;\frac{1}{A_{disc}},$$

where *m* is mass (μg), *t* is time (min), *A_disc_* is the disc surface area (cm^2^), *V* is the volume of the medium (mL), and *k* is the slope of the straight line from the dissolution profile (μg/(min × mL)). Intrinsic dissolution rates will henceforth be denoted ‘IDR’ for both SIDR and DIDR values determined in this study (see Table 3 for details).

### 2.5. Statistics and Physicochemical Analysis

All IDR data are presented as mean values ± standard deviation (n ≥ 3). For clarity, only selected data points are shown in the dissolution curves, however, all data points obtained for the dissolution under sink conditions were used for the calculations. Univariate analyses of physicochemical properties and their relation to IDR was performed in Excel. Multivariate data analysis in the form of partial least squares projection to latent variables (PLS) was performed in Simca-P 11.0 (Umetrics, Umeå, Sweden). For the latter, only non-skewed physicochemical descriptors (calculated by ADMET Predictor 9.0, Lancaster, CA, USA) were included and these were used after being mean centered and scaled to unit variance. Because the dataset still is relatively small for a PLS analysis, these tests were performed to identify trends rather than provide quantitative analyses. A variable selection was performed where variables without significant influence on the response (log IDR) were excluded from the model. The variable selection was performed step-wise where the least important variable according to the variable importance in projection (VIP)-plot was excluded, and the modeling repeated. The influence on the model was studied by following the cross—validated R^2^ (Q^2^), making use of seven leave-one-out groups. When exclusion of the least important variable was found to increase predictivity (as assessed by increased Q^2^), that particular variable was permanently left out of the model. The variable selection was repeated until no further improvement in Q^2^ could be obtained. Model validity was also checked by performing a permutation test making use of 100 iterations. Using this approach, it was possible to make a qualitative investigation of which properties are most closely linked to IDR in biorelevant media containing bile salts and phospholipids.

## 3. Results and Discussion

### 3.1. Rapid Preparation of Controlled Suspensions by Ultrasonication and their Characterization

Andersson et al. [7] developed a small-scale dissolution method using controlled suspensions to rapidly determine IDR of poorly water-soluble compounds in phosphate buffer and fasted-state biorelevant medium. In that study, the controlled suspensions were prepared by ball milling APIs for 20 min in an aqueous medium containing a low concentration (1.0% w/w) of a surfactant for (primary) particle stabilization, and these suspensions were then used for small-scale dissolution measurements in the μDiss Profiler. The protocol established by Andersson et al. was used to study six poorly water-soluble compounds. Here, we developed this method further with the aims of (i) establishing an experimental protocol for rapid preparation of suspensions that in turn would allow (ii) including solid-state analyses of the suspensions, as well as (iii) measuring the dissolution rate of a larger dataset of compounds in several different biorelevant media. Our expanded method was evaluated with a diverse set of compounds (including acids, bases, and nonionizable compounds) to explore its general applicability for IDR measurements in simulated intestinal fasted and fed state dissolution media. Thus, we focus on understanding dissolution of solid materials (either from the dosage form or by API precipitation) in the small intestine, as the majority of drug absorption takes place there. We explore the fed state, since it is well-known that the additional amount of lipids and bile salts in the dissolution medium may significantly increase solubilization and hence, the total amount of API dissolved. An additional goal was to investigate relationships between physicochemical properties of the APIs and their IDRs. The dataset assembled in this study is the largest IDR library available in the open literature to date (Table 3).

Controlled suspensions were prepared for all compounds with S < 100 μg/mL following the procedures of Andersson et al. [17]. The disc method was applied for compounds with higher solubility values (primarily acids). An exception was ethinylestradiol, which had an unexpectedly rapid dissolution (so rapid that IDR could not be calculated making use of suspension) and thus the disc method was used. We found that ultrasonication instead of ball milling was a very efficient method for dispersing the compounds in the suspension medium, and the preparation time could be reduced to a maximum of five minutes compared to the considerably longer time (20 min) required for ball milling [7]. Furthermore, ultrasonication allows a more efficient use of compound, while ball milling requires relatively large amounts of API. With ball milling, some amount of compound is lost to the apparatus because it is difficult to extract the whole suspension volume from the ball mill. In addition, sieving is required at times to separate milling beads from the suspension, leading to further loss of API.

The DLS peak intensities of the suspensions prepared for dissolution in FaSSIF and FeSSIF are listed Table S1 (Supplementary Materials). Most compounds had monomodal particle size distributions with peak intensities of about 1–2 μm, in good agreement with ball milled suspensions [7]. Bimodal particle size distributions were obtained for a few compounds, typically with a smaller peak appearing at about 100–200 nm. In these cases, both particle populations were used for calculating the total particle surface area in suspension. Ideally, the particle size in strongly bimodal suspensions should be measured by laser diffraction; however, that measurement method requires significantly larger sample volumes, that might not be feasible to produce during the early drug development stage. Two compounds needed slightly different treatment: felodipine and griseofulvin. Felodipine had a monomodal size distribution after 1 min of ultrasonication (Table S1, used for dissolution measurements) similar to ball milling [7], while continued ultrasonication for 5 min resulted in a bimodal size distribution (Figure S1, Supplementary Materials). Griseofulvin dispersed well in the suspension medium by vortexing, resulting in a single mode of particle size distribution at 1.4 μm (Table S1). We used this vortexed suspension for dissolution measurements because ultrasonication for just one minute resulted in a bimodal size distribution for griseofulvin, (Figure S1, Supplementary Materials). This had also been observed previously with ball milling [7]. In general, particle size in the suspensions could vary from sample to sample for each compound due to the inhomogeneity of the as-received, coarse starting materials. However, this is accounted for in the protocol, as the suspension size of each prepared suspension was measured by DLS and the corresponding surface area was used for the subsequent IDR calculations.

An advantage of the rapid preparation time of suspensions by ultrasonication compared to ball milling is the reduced risk of solid phase transformations occurring during processing. It has been shown that ball milling can induce a gradual amorphization which in turn can alter the dissolution rate of the compound [51]. All compounds received were found in their thermodynamically most favorable polymorphs (Table 1). A comparison of XRD patterns before and after ultrasonication revealed no polymorphic changes for any of the compounds (Figures S2–S4, Supplementary Materials) except for carvedilol. The XRD pattern of as-received carvedilol powder corresponded to previously reported form II with a main diffraction peak at 2Θ 17.5° [24,25,52]. The carvedilol suspension had main XRD peaks at 2Θ 26.4, 23.2, and 16.1°. This spectrum could not be clearly attributed to previously known carvedilol polymorphs, but could be a mixture of the as-received form II and a hemihydrate [25].

### 3.2. Dissolution Profiling

**Figure 1:**
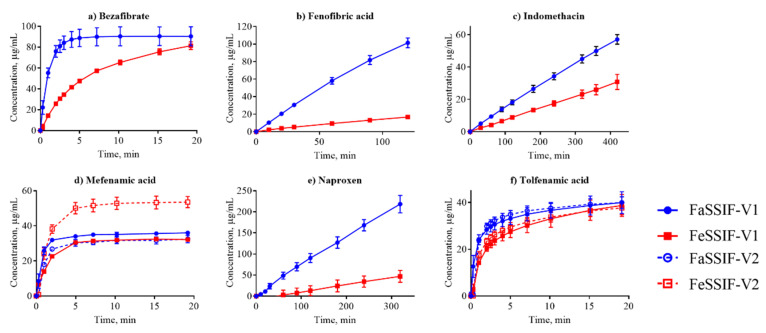
Dissolution profiles of the acidic compounds in fasted state simulated small intestinal fluid (FaSSIF) (circles, blue lines) and fed state simulated small intestinal fluid (FeSSIF) (squares, red lines). Dissolution of fenofibric acid, indomethacin, and naproxen were measured from discs, while bezafibrate, mefenamic acid, and tolfenamic acid were suspension assays. The dissolution profiles from the discs were acquired over several hours due to the small disc surface area, while suspension measurements were completed within 20 min.

**Figure 2:**
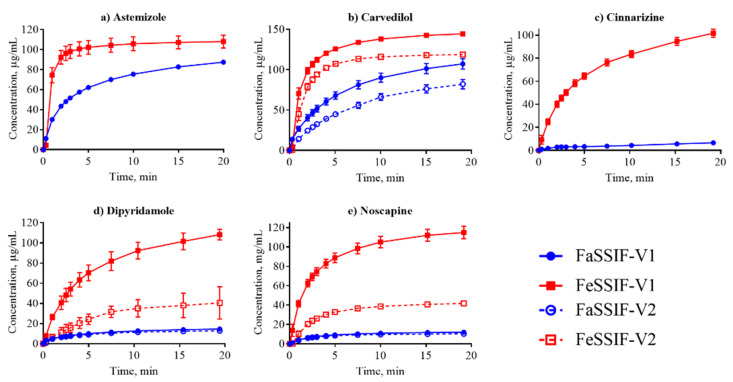
Dissolution profiles of the basic compounds from their suspensions in FaSSIF (circles, blue lines) and FeSSIF (squares, red lines).

**Figure 3:**
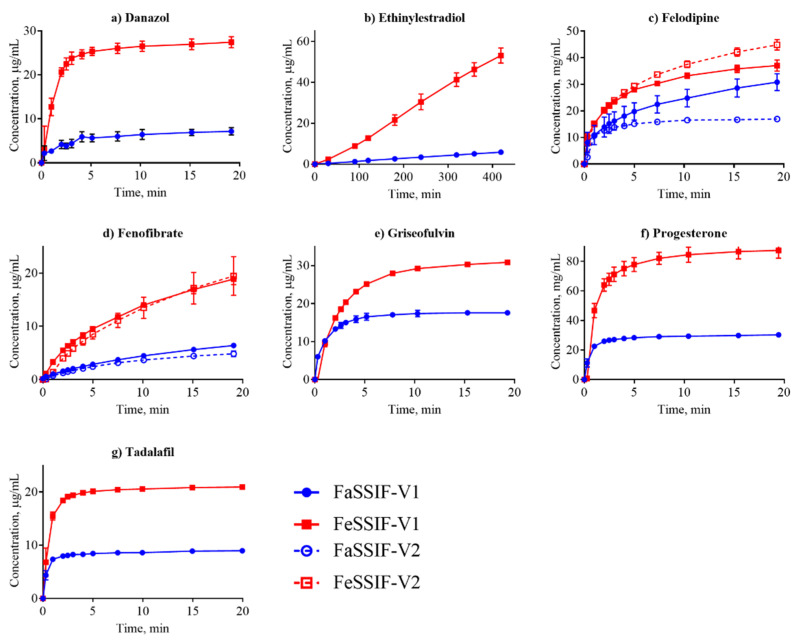
Dissolution profiles of the nonionized compounds in FaSSIF (circles, blue line) and FeSSIF (squares, red line). Ethinylestradiol dissolution was determined from a disc; all other compounds were measured from suspensions.

All IDR data that we determined and that are discussed in this section are shown in Table 3 with the corresponding dissolution profiles in Figure 1, Figure 2 and Figure 3 in FaSSIF and FeSSIF for acidic, basic and nonionized compounds, respectively. We compared our absolute IDR values to values available in the literature in order to validate the proposed SIDR method. Andersson et al. [7] reported SIDR values in FaSSIF-V1 for cinnarizine, felodipine, fenofibrate, and tadalafil, and those values were in good agreement (R^2^ = 0.78) with the values determined in this study, demonstrating the robustness of our method, despite methodological differences in both suspension preparation and the dissolution assay itself. Andersson et al. [7] showed that the saturation level (or excess) of compound added to the dissolution medium has limited effect on the determined SIDR. Our data confirms that result, as the added drug concentrations were kept constant for all compounds in all media (129 μg/mL) resulting in different saturation ratios compared to the respective S_app_ but nevertheless yielding comparable results. In this study, no compensations for dilution effects were made (regarding, e.g., taurocholate and lecithin concentrations [7]) because we wanted to provide as streamlined a method as possible, and our results justify this decision.

Dissolution profiles in the FaSSIF-V2 and FeSSIF-V2 media could only be established for seven of the compounds (Figure 1, Figure 2 and Figure 3): mefenamic acid, tolfenamic acid, carvedilol, dipyridamole, noscapine, felodipine, and fenofibrate. Additionally, astemizole, ethinylestradiol, naproxen, and tadalafil could be measured in FaSSIF-V2 (Table 3). Several issues prevented IDR determination in these V2 media for the other compounds: either standard curves could not be established or dissolution curves were highly scattered and non-reproducible. The second version of the biorelevant media were developed originally to more closely mimic the composition of the human intestinal environment based on *in vivo* data [53]. The buffer system was changed from phosphate buffer in FaSSIF-V1 to a maleate buffer in FaSSIF-V2 and FeSSIF-V2 (Table 2) to reflect the physiologically relevant buffer capacity and osmolarity [54]. However, maleic acid exhibits a strong UV absorbance <250 nm, which prevents analysis of compounds with UV detection wavelengths <300 nm. Due to the remaining small IDR dataset in FaSSIF-V2 and FeSSIF-V2, we primarily focused our following data analysis on the IDR dataset compiled in FaSSIF-V1 and FeSSIF-V1.

**Figure 4:**
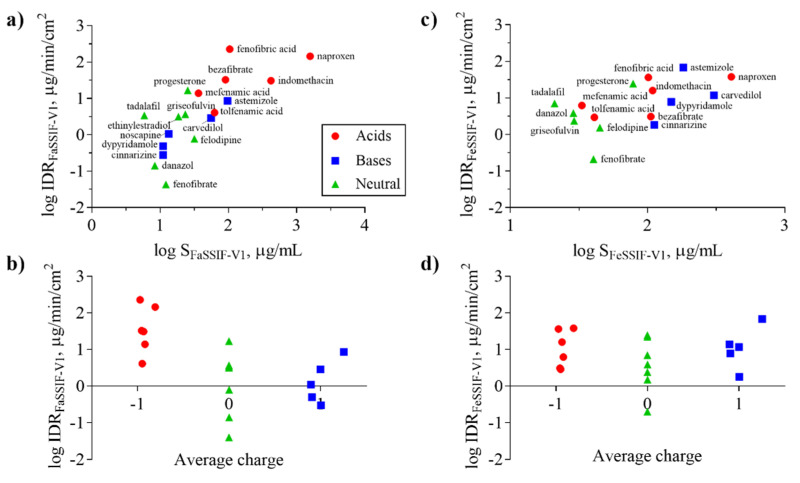
Intrinsic dissolution rate (IDR) as a function of solubility (S) (**a**,**c**) and average charge (**b**,**d**) for acids (red symbols), bases (blue symbols), and neutral (green symbols) compounds in FaSSIF-V1 (**a**,**b**) and FeSSIF-V1 (**c**,**d**). Average charge represents the net formal negative charge of the molecules (QAvgNeg) at pH 6.5 (**b**) and 5 (**d**), respectively. Ionization of the compounds in the respective media (FaSSIF-V1 pH 6.5; FeSSIF-V1 pH 5) clearly shows that acids have higher IDR in FaSSIF-V1 than bases, whereas the separation of bases displaying higher IDR in FeSSIF-V1 is not as clear.

Figure 4 shows IDR as a function of S in FaSSIF-V1 (a) and FeSSIF-V1 (c), respectively. It is clear that higher solubility correlates in general with higher IDR in both media which is described by the classic Noyes-Whitney equation [55] relating dissolution rate to solubility and diffusion:

(8) $$\frac{dC}{dt}=\frac{D}{h}\;A\;(C_{S}-C_{t}),$$

where *dC*/*dt* is the change in concentration over time (i.e., dissolution rate), *D* is the diffusion coefficient (cm^2^/s), *h* is the thickness of the diffusion layer (cm), *A* is the surface area (cm^2^), *C_s_* is the saturated concentration (i.e., the thermodynamic solubility), and *C_t_* is the concentration of the dissolved compound in the bulk at time *t*. Thus, a higher solubility will lead to higher *C_t_* and an increased dissolution rate.

The solubility of ionizable compounds increases with charge and is therefore pH-dependent as described by the Henderson-Hasselbalch equation [56]. Acids are ionized to a higher degree and have a higher solubility in FaSSIF-V1 (pH 6.5) than FeSSIF-V1 (pH 5), while the opposite holds for bases (Figure 4b,d). Our ordering of IDR values, namely acids > bases for IDR_FaSSIF-V1_ is the result of compound ionization (Figure 4b). Similarly, all bases have a significantly higher IDR values in FeSSIF-V1 compared to FaSSIF-V1 due to their ionization. The pH-dependent solubility and as a result, its effect on IDR, was also confirmed for the two basic compounds dipyridamole and noscapine that could be profiled also in FaSSIF-V2 and FeSSIF-V2 (Figure 2). Here, IDR were comparable in FaSSIF-V1 and FaSSIF-V2, while higher IDR values were found in FeSSIF-V1 compared to FeSSIF-V2, respectively (Table 3). This can be attributed to the higher pH (Table 2) in FeSSIF-V2 (pH 5.8) compared to FeSSIF-V1 (pH 5) and thus follows the pH-dependent compound ionization. Similarly, for mefenamic acid and tolfenamic acid, the lowest IDRs in the four biorelevant media studied were found in FeSSIF-V1 due to their low ionization at pH 5 in this medium. Overall, comparing acids and bases, the separation in their IDR values is not as clear in FeSSIF-V1 compared to FaSSIF-V1 (Figure 4).

However, IDR is not solely governed by compound solubility and its dependence on pH because it can be seen that compounds with similar S have notably different IDR values (Figure 4a,c). For example, fenofibrate, cinnarizine, dipyridamole, and noscapine all have comparable S in FaSSIF-V1 (11–13 μg/mL; Table 1), but their IDR-values differ significantly (note the log scale in Figure 4). We observed similar trends in FaSSIF-V1 for astemizole, bezafibrate, and fenofibric acid, a group of compounds with S ~ 100 μg/mL (Table 1), and in FeSSIF-V1 for cinnarizine, bezafibrate, indomethacin, and fenofibric acid.

### 3.3. Impact of Physicochemical Properties on IDR

We analyzed the FaSSIF-V1 and FeSSIF-V1 IDR results in more detail to see whether any correlations between IDR and other chemical properties could be detected. Univariate statistical analysis did not show a strong correlation between individual physicochemical properties (e.g., M_w_, log D, rotatable bonds, hydrogen bond donors/acceptors) and IDR. The predicted diffusion coefficients of the API molecules in water was not linked to IDR for this dataset, in contrast to expectations based on dissolution theory (i.e., the Noyes-Whitney equation).

Our dataset included some relatively lipophilic compounds with a logD_pH6.5_ ranges of 1.0–5.2 (mean 2.8), and such compounds are likely to partition significantly into micelles formed by bile salts and phospholipids in the biorelevant dissolution media [57]. The effective diffusion (*D_eff_*) for extensively solubilized drugs is a result of the molecular and the micelle diffusion, as described by the following equation:

(9) $$D_{eff}=D_{u}f_{u}+D_{m}f_{m},$$

where *D_u_* and *D_m_* are the average diffusion coefficients of the unbound drug and the micelle, respectively, and *f_u_* and *f_m_* are the fraction of drug being unbound and bound to micelles, respectively [58,59]. Interactions with micelles is governed by hydrophobic and electrostatic interactions between compound and micelles, but other factors, such as micelle size and shape, can also play a role for compound insertion into lipid aggregates [60]. Micelles hold a negative net charge and thus favor interactions with cations and neutral compounds over interactions with anions.

Dissolution is often considered in terms of “brick dust molecules” versus “grease balls” metaphors [61]. These concepts are described by the General Solubility Equation, which accounts for the compound melting point and lipophilicity [62]. “Brick dust molecules” are compounds with a T_m_ >200°C, and their dissolution can be limited by the high energy required to dissociate molecules from their solid crystal lattice (i.e., solid-state limitation). In this study danazol, griseofulvin, mefenamic acid, tadalafil and tolfenamic acid are considered to be “brick dust” compounds based on their high T_m_ (Table 1). In contrast, “grease ball” compounds are poorly hydrated due to their high lipophilicity (log D > 3), resulting in solvation-limited solubility. Of the above-mentioned solid-state limited compounds, danazol is also regarded as a solvation-limited compound. From a dissolution perspective, danazol is likely to have slow dissociation from its crystal lattice, but once dissociation occurs, danazol molecules partition rapidly into micelles in the media. We observe that IDR for danazol increases 28-fold in the fed-state medium compared to fasted-state (Table 3), which would be predicted because fed-state has a much greater volume fraction of micelles. This result suggests that the solvation-limiting factor is more important than the dissociation from the crystal lattice. In contrast, griseofulvin and tadalafil, both being ‘true’ brick dust molecules with logD < 3, displayed lower (griseofulvin) or double (tadalafil) IDR in FeSSIF-V1. These results suggest that the dissociation from the crystal lattice is more important for these two molecules than the solvation limitation.

We compared our IDR results in FaSSIF and FeSSIF with the recommendations of food intake for oral administration of these drugs (Table 1). Food or specially designed formulations (e.g., lipid-based formulations) can increase the absorption of poorly water soluble compounds. For instance, fenofibrate should be taken with food as it increases bioavailability, in line with the higher solubility and IDR (Figure 3d, Table 3) measured here. Fenofibrate is considered a “grease ball” molecule with a low melting temperature (Table 1), and its solvation thus greatly benefits from the presence of lipids. Basic compounds, with the exception of astemizole and noscapine, should be taken with food (Table 1), as their solubility and IDR values increase in the fed state. Examples are cinnarizine and carvedilol, with a higher solubility and IDR in FeSSIF than in FaSSIF observed here, and the positive food effect also recently reported in literature [63,64]. All compounds with no reported food effect are either neutral or acids (Table 1) in agreement with our IDR data. It should be noted, that recommendation to take compounds with food can also be given to slow down absorption (but not necessarily the extent of it) and thereby reduce side effects (e.g., for bezafibrate, carvedilol, dipyridamole and indomethacin). Thus food recommendations cannot in all cases be correlated solely to compound solubility and IDR.

**Figure 5:**
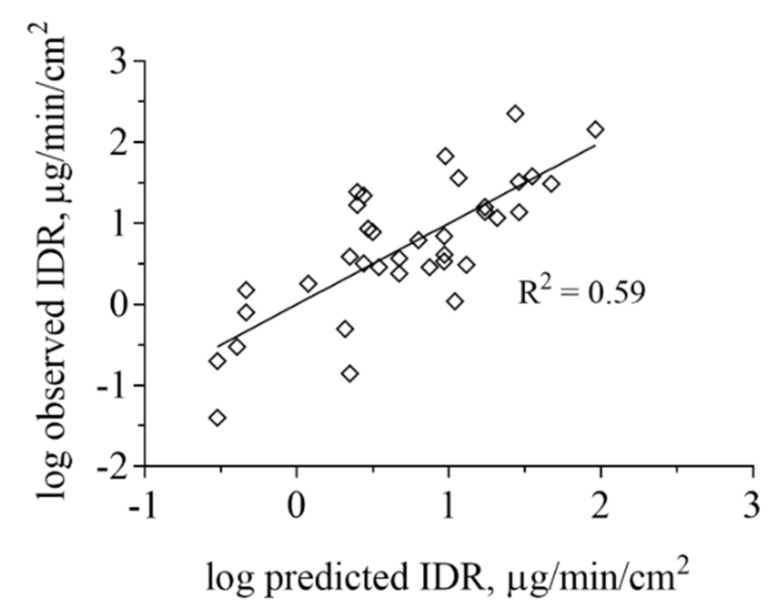
Projection to latent variables (PLS) regression analysis based on ADMET Predictor of IDR measured in FaSSIF-V1 and FeSSIF-V1. The lipophilicity and ionization were the most important properties governing the IDR of this dataset but also electron density, as described by the Maximum sigma Fukui index was found to contribute to the IDR values obtained. IDR in biorelevant media based on phospholipids and taurocholate is strongly determined by API distribution into micelles for lipophilic, poorly water-soluble compounds.

We also performed multivariate statistics to look for correlations between IDR and other physicochemical properties. PLS regression analysis based on calculated molecular physicochemical properties confirmed that ionization in combination with drug lipophilicity are important factors influencing IDR (Figure 5). A variable selection resulted in four descriptors being the main properties governing the IDR of this dataset. These were reflecting, in order of importance, lipophilicity (logD), negative charge (fraction anionised at specific pH (F_anion_), net formal negative charge at specific pH (QAvgNeg)) and electron density (maximum sigma Fukui index on C). Model statistics indicate that this is a qualitative model and that the descriptors cannot be used to quantitatively predict IDR (R^2^ of 0.59). All data points were used for the model development and for that reason, the model has not been subjected to an external test set. However, neither the leave-one-out cross-validation (Q^2^ of 0.53) nor the permutation test showed signs of the model being overfitted.

We conclude that the IDR values in biorelevant media that contain phospholipids and taurocholate depend strongly on partitioning into formed micelles, and that this partitioning depends on lipophilicity and ionization. In fact, these considerations align with the recently proposed indirect loading mechanism of dissolving drug molecules into FaSSIF micelles [65,66], which suggests that the equilibrium partition coefficient of the API between the micellar phase and the buffer phase can prevent dissolved drug molecules from loading a micelle up to its equilibrium saturated value. This results in slower dissolution rates than predicted by the Noyes-Whitney equation, and has been observed experimentally for danazol in FaSSIF [60,65]. Clearly, further exploration of molecular properties important for IDR would benefit from computational modeling of dissolution, which would require even larger datasets than the one established here. It would also be interesting to explore other computational and experimental techniques, e.g., Molecular Dynamics simulations [67] or small angle X-ray scattering (SAXS) analyses of API partitioning, into these micelles [68,69], which could suggest ways for fine-tuning computational modeling. Finally, the IDR data produced herein can be used to obtain better physiology-based pharmacokinetics (PBPK) models after oral administration of, e.g., formulations containing these compounds.

## 4. Conclusions

This study reports, for the first time in open literature, a large dataset of the IDR values of compounds with diverse physicochemical properties, in both fasted (FaSSIF) and fed state simulated intestinal fluids (FeSSIF). The refined control suspension method developed here, making use of ultrasonication, effectively disperse API particles in suspension media within a few minutes, without inducing solid state transformations that could affect dissolution rates. For the 18 compounds studied in FaSSIF and FeSSIF, we found that IDR cannot be predicted solely from solubility; in fact no single physicochemical property was found that could reliably predict IDR. Ionization was weakly correlated to IDR in acids, and the acids were furthermore found to exhibit higher IDR compared to bases especially in FaSSIF. When relating combined molecular descriptors to the IDR data the importance of lipophilicity, ionization, and electrostatic interactions for the compounds partitioning into phospholipid and bile salt micelles was revealed. The presented medium throughput method could greatly expand the IDR database and is highly applicable to an industrial setting, making it possible to measure at least 150 compounds in single runs (or 50 compounds in triplicate) per week. Having a more extensive IDR database with high quality data corrected for potential solid state changes would enable the ultimate goal—to predict IDR *in silico* based solely on molecular descriptors of the API.

## Figures and Tables

**Table 1:** Physicochemical properties of active pharmaceutical ingredients (APIs).

Compound	Structure	M_w_^*^ g/mol	A/B/N	pK_a_	logP	T_m_ °C	ΔH_f_ J/g	Crystalline Form As-Received^†^	Food ^‡^
Astemizole	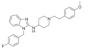	458.6	B	8.1	5.9	174 [22]	111 [22]	(ZENREP)*	-
Bezafibrate	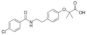	361.9	A	3.5	4.0	182	145	α form [23]	+
Carvedilol	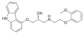	406.5	B	8.1	4.0	117 [24]	120 [24]	form II [25]	+
Cinnarizine	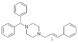	368.6	B	7.4	5.3	117	118	([26])	+
Danazol	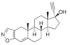	337.5	N	-	3.6	226	92	([27])	+/-
Dipyridamole	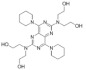	504.7	B	6.8; 3.6	2.2	166 [28]	56 [28]	form II [29]	+
Ethinylestradiol	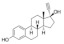	296.4	N	-	3.6	183 [30]	89 [31]	hemihydrate [31]	+/-
Felodipine	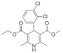	384.3	N	-	4.8	142	76	form I [32]	+/-
Fenofibrate	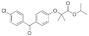	360.9	N	-	5.3	79	98	form I [33]	+
Fenofibric acid	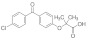	318.8	A	3.5	4.0	181 [34]	-	(QANHUJ)*	+/-
Griseofulvin	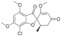	352.8	N	-	2.5	215	122	([35])	+
Indomethacin	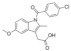	357.8	A	4.1	3.8	160 [22]	106 [22]	γ form [36]	+/-
Mefenamic acid	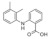	241.3	A	4.0	4.9	230 [37]	158 [37]	form I [38]	+
Naproxen	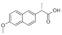	230.3	A	4.5	3.3	156 [22]	149 [22]	form I [39]	+/-
Noscapine	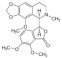	413.4	B	6.1	2.8	176 [40]	-	([40])	-
Progesterone	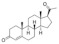	314.5	N	-	3.9	129 [41]	89 [42]	form I [43]	+
Tadalafil	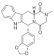	389.4	N	-	1.6	295 [44]	113 [44]	form I [45]	+/-
Tolfenamic acid	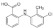	261.7	A	4.2	5.3	212	143	form I [46]	n/a

M_W_: molecular weight; A: acid, B: base, N: neutral in the pH range 2–12; pK_a_: acidic dissociation constant calculated with ADMET Predictor; logP: partition coefficient between octanol and water calculated with ADMET Predictor (S+logP); T_m_: melting point determined at onset of melting; ΔH_f_: enthalpy of fusion. ^†^ References are given in brackets, when only one polymorphic form of the compound is known. * Reference code from Cambridge Structure Database. ‡ Recommendations for oral administration from Drugbank.ca and from the Swedish Physician’s Desk Reference (fass.se), +/-: no food effect, +: take with meal, -: take between meals.

**Table 2:** Composition of the biorelevant media [47], pH, and hydrodynamic diameter.

Component		FaSSIF-V1	FeSSIF-V1	FaSSIF-V2	FeSSIF-V2
Taurocholate	mM	3	15	3	10
Phospholipids		0.75	3.75	0.2	2
Sodium		148	319	106	218
Chloride		106	203	69	125
Phosphate		29			
Acetic acid			144		
Maleic acid				19	55
Oleate					0.8
Glycerol monleate					5
**pH**		6.5	5	6.5	5.8
**Hydrodynamic diameter**	nm	71	8	32	35

**Table 3:** API apparent solubility, experimentally determined intrinsic dissolution rates (IDR) from disc (D) or suspensions (S) of the compounds in fasted (FaSSIF-V1 and FaSSIF-V2) and fed (FeSSIF-V1 and FeSSIF-V2) state simulated intestinal fluids.

Compound	S_FaSSIF-V1_ μg/mL	S_FeSSIF-V1_ μg/mL	D/S	IDR_FaSSIF-V1_ μg/min/cm^2^	IDR_FeSSIF-V1_ μg/min/c^2^	IDR_FaSSIF-V2_ μg/min/cm^2^	IDR_FeSSIF-V2_ μg/min/cm^2^
Astemizole	97.9 ± 2.1 [15]	182 ± 1 [15]	S	8.6 ± 0.4	67.5 ± 14	2.0 ± 0.2	-
Bezafibrate	91.0 ± 8.9 †	105.7 ‡	S	32.7 ± 3.9	3.1 ± 0.04	-	-
Carvedilol	55.9 ± 1.0 [15]	305 ± 2 [15]	S	2.9 ± 0.5	11.7 ± 0.9	1.5 ± 0.2	13.6 ± 0.6
Cinnarizine	11.1 ± 1.2 [7]	112 ± 2 [15]	S	0.3 ± 0.01	1.8 ± 0.07	-	-
Danazol	8.4 ± 0.7 [15]	28.8 ± 0.4 [15]	S	0.14 ± 0.02	3.9 ± 0.4	-	-
Dipyridamole	11.1 ± 3.0 [48]	148.9 ± 19.7 [48]	S	0.5 ± 0.05	7.8 ± 2.6	0.5 ± 0.03	1.7 ± 0.3
Ethinylestradiol	18.7 ± 1.3 *	-	D	3.2 ±0.1	22.1 ±3.1	6.6 ± 0.8	-
Felodipine	31.8 ± 1.7 [7]	44.9 ± 9.2 [48]	S	0.8 ± 0.4	1.5 ± 0.06	0.6 ± 0.2	1.5 ± 0.2
Fenofibrate	12.2 ± 0.3 [7]	40.4 ± 2.9 [49]	S	0.04 ± 0.002	0.2 ± 0.008	0.05 ± 0.001	2.7 ± 0.3
Fenofibric acid	104.8 ± 1.0 †	101.4 ± 3.8 †	D	227 ± 33	36.3 ± 5.8	-	-
Griseofulvin	23.4 ± 1.8 [41]	29.2 ± 3.4 [41]	S	3.7 ± 0.6	2.4 ± 0.02	-	-
Indomethacin	421.7 ± 17.6 [7]	109.0 ± 7.0 [15]	D	31 ± 1.3	16 ± 1.0	-	-
Mefenamic acid	36.6 ± 0.6 †	33.2 ± 0.7 †	S	13.9 ± 2.0	6.2 ± 0.9	11.9 ± 1.0	7.9 ± 2.1
Naproxen	1584.8 ± 101.4 *	408.0 ± 35.1 *	D	144 ± 12	38 ± 1.5	179 ± 11	-
Noscapine	13.4 ‡	-	S	1.1 ± 0.2	13.8 ± 1.3	1.2 ± 0.1	5.2 ± 0.8
Progesterone	25.5 ± 1.1 [50]	78.6 ± 16.2 [41]	S	16.8 ± 0.5	24.5 ± 5.7	-	-
Tadalafil	5.9 ± 0.7 [7]	21.0 ± 0.5 †	S	3.4 ± 0.7	7.0 ± 0.9	1.3 ± 0.8	-
Tolfenamic acid	63.0 ± 2.7 [15]	41.0 ± 0.5 [15]	S	4.1 ± 0.8	2.9 ± 0.2	12.9 ± 0.9	4.8 ± 0.6

* Solubility determined by shake-flask method at 37 °C and high-performance liquid chromatography (HPLC) analysis. † Solubility values determined from dissolution experiments in the μDiss Profiler. ‡ Solubility values determined by extrapolation of dissolution data in the μDiss Profiler; nonlinear regression analysis in GraphPad Prism 7.04.

## Supplementary Materials

The following are available online at https://www.mdpi.com/1999-4923/12/6/493/s1, Figure S1: Particle size distribution measured by dynamic light scattering (DLS) for griseofulvin (a) and felodipine (b) as a function of ultrasonication time. Figure S2: X-ray diffraction patterns of the acidic compounds as-received, as well as after preparation of their suspensions. Figure S3: X-ray diffraction patterns of the bases as-received, as well as after preparation of their suspensions. Figure S4: X-ray diffraction patterns of the neutral compounds as-received, as well as after preparation of their suspensions. Table S1: Peak intensity of API suspensions prepared for subsequent dissolution analysis in the biorelevant media.
