# Supplementary material for: Intrinsic Dissolution Rate Profiling of Poorly Water-Soluble Compounds in Biorelevant Dissolution Media
Source: Pharmaceutics. 2020 May 28;12(6):493. doi: 10.3390/pharmaceutics12060493 (PMC7356998; doi:10.3390/pharmaceutics12060493)
Supplement: Supplementary file 1 [file pharmaceutics-12-00493-s001.pdf]

pharmaceutics

Supplementary Materials

Alexandra Teleki [1,*], Olivia Nylander [2] and Christel A.S. Bergström [2,3,*]

[1] Science for Life Laboratory, Department of Pharmacy, Uppsala University, Uppsala Biomedical Center P.O. Box 580, SE-75123 Uppsala, Sweden
[2] Department of Pharmacy, Uppsala University, Uppsala Biomedical Center P.O. Box 580, SE-75123 Uppsala, Sweden; olivianylander@gmail.com
[3] The Swedish Drug Delivery Center, Department of Pharmacy, Uppsala University, Uppsala Biomedical Center P.O. Box 580, SE-75123 Uppsala, Sweden
* Correspondence: alexandra.teleki@farmaci.uu.se (A.T.); christel.bergstrom@farmaci.uu.se (C.A.S.B.); Tel.: +46–18-471 4745 (A.T.); +46-18-471 4118 (C.A.S.B.)

Table S1. Peak intensity of API suspensions prepared for subsequent dissolution analysis in the biorelevant media.

Compound	Peak intensity DLS nm			
	**FaSSIF-V1**	**FeSSIF-V1**	**FaSSIF-V2**	**FeSSIF-V2**
Astemizole	1558	1558	-	-
Bezafibrate	2438	1290	-	-
Carvedilol	748	748	822/46	1045/9626/71
Cinnarizine	1392	1938/187	-	-
Danazol	1102/149	1226/113	-	-
Dipyridamole	982	1926	982	1926
Ethinylestradiol	-	-	-	-
Felodipine	824	1017	824	1019
Fenofibrate	5245/461	3920/334	5245/461	3920/334
Fenofibric acid	-	-	-	-
Griseofulvin	1365	1365	-	-
Indomethacin	-	-	-	-
Mefenamic acid	1702	2184	1702	2184
Naproxen	-	-	-	-
Noscapine	1411	1494	1411	1494
Progesterone	1926	1926	-	-
Tadalafil	1320/203	1182/194	1320/203	-
Tolfenamic acid	1136/242	1076/143	1136/242	1076/143

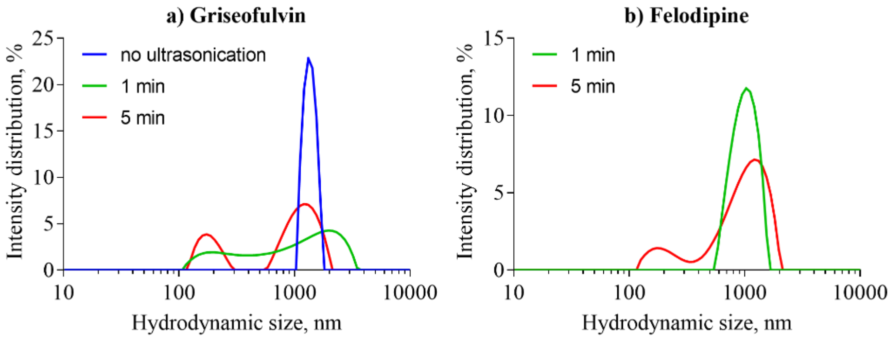

Figure S1. Particle size distribution measured by dynamic light scattering (DLS) for griseofulvin (**a**) and felodipine (**b**) as a function of ultrasonication time.

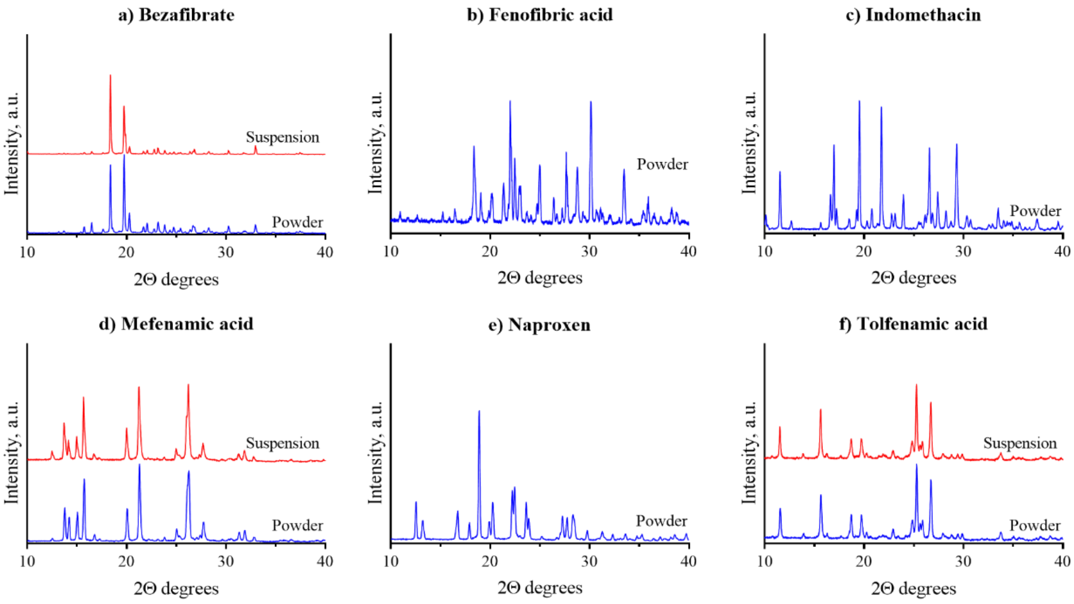

Figure S2. X-ray diffraction patterns of the acidic compounds as-received (blue line) as well as after preparation of their suspensions (red line). Fenofibric acid, indomethacin and naproxen were used in disc assays and thus only their powder XRD is shown.

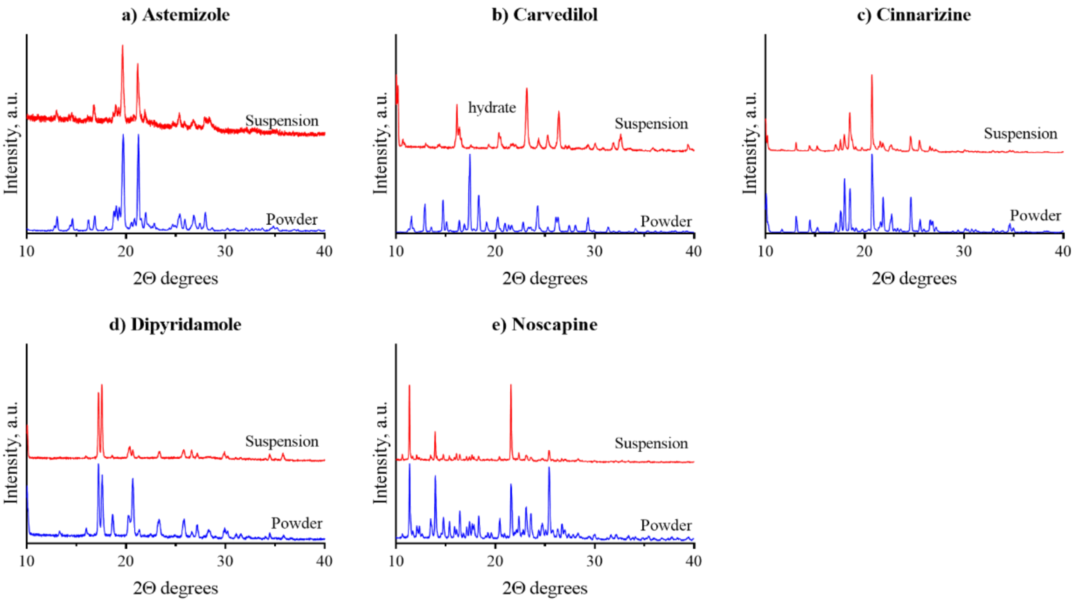

Figure S3. X-ray diffraction patterns of the bases as-received (blue line) as well as after preparation of their suspensions (red line).

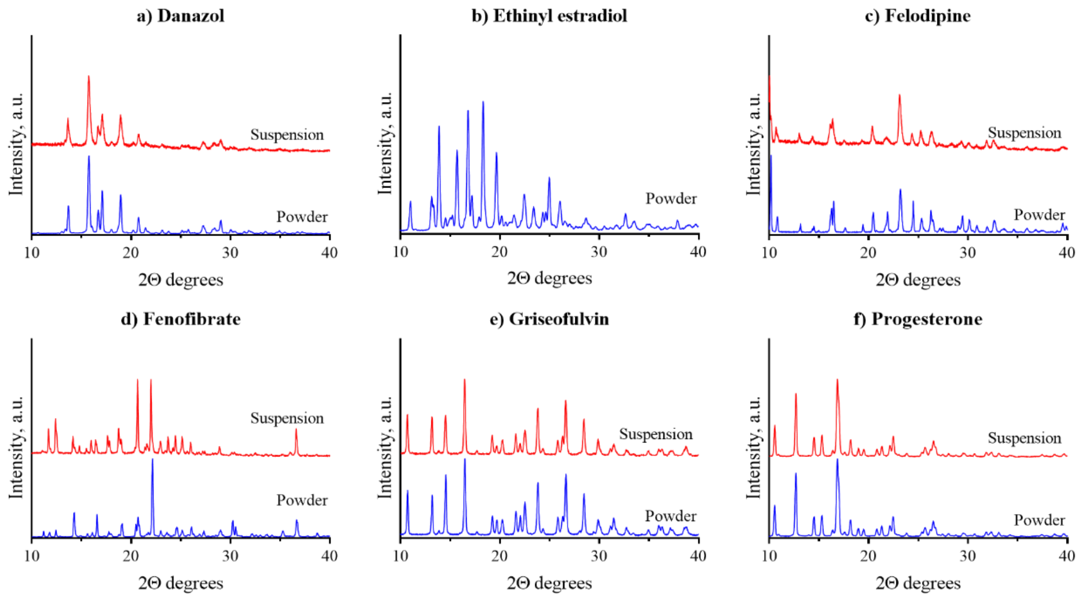

Figure S4. X-ray diffraction patterns of the neutral compounds as-received (blue line) as well as after preparation of their suspensions (red line). Ethinylestradiol was used in disc assays and thus only its powder XRD is shown.
